# Hemodynamic subtype profiling in tremor-dominant and akinetic–rigid Parkinson’s disease using multi-delay pseudo-continuous arterial spin labeling imaging

**DOI:** 10.3389/fnhum.2025.1617996

**Published:** 2025-07-02

**Authors:** Yu Pan, Zhaoxia Qin, Jiangbing Liu, Wei Wang, Yi Zhao

**Affiliations:** ^1^Department of Medical Imaging, Affiliated Hospital of Yangzhou University, Yangzhou, China; ^2^Department of Neurology, Affiliated Hospital of Yangzhou University, Yangzhou, China

**Keywords:** Parkinson’s disease, tremor, rigidity, MD-pCASL, hemodynamic

## Abstract

**Purpose:**

This study investigated the differences in global cerebral hemodynamics between the tremor-dominant (TD) and akinetic–rigid-dominant (ARD) subtypes of Parkinson’s disease (PD) using multi-delay pseudo-continuous arterial spin labeling (MD-pCASL) imaging and evaluated the clinical value of MD-pCASL for identifying PD subtypes.

**Methods:**

Twenty-five healthy controls (HC) and fifty-one patients with PD were enrolled: 26 in the TD group and 25 in the ARD group. Voxel-based analysis was performed to compare cerebral blood flow (CBF), arterial transit time (ATT), and cerebral blood volume (CBV) among the ARD, TD, and HC groups. Binary logistic regression analysis was used to screen independent influencing factors for predicting motor subtypes; a receiver operating characteristic curve was drawn to evaluate the diagnostic value of the multi-parameter arterial spin labeling (ASL) technique.

**Results:**

Compared with the HC group, the ARD group exhibited increased CBF in the left thalamus, lingual gyrus, and hippocampus. Decreased CBF was observed in the left angular gyrus. The TD group exhibited decreased CBF in the left precuneus compared with that in the HC group. Compared with the TD group, the ARD group exhibited increased CBF in the left putamen, lingual gyrus, and hippocampus. Differences in CBV mirrored CBF trends. ATT prolongation was observed in the left middle temporal gyrus in the ARD group. Diagnosing ARD as positive, the following were considered potential risk factors: increased CBF in the left putamen and left lingual gyrus, increased CBV in the left lingual gyrus, and prolonged ATT in the left middle temporal gyrus. The combined areas under the curve of all indexes were 0.942; the optimal critical value was 0.766; sensitivity was 92%; and specificity was 84.6%.

**Conclusion:**

Both subtypes exhibited hypoperfusion patterns predominantly in the parieto-occipital cortex, whereas patients with ARD uniquely displayed hyperperfusion within the basal ganglia nuclei. Moreover, multi-parameter ASL showed high diagnostic efficiency in distinguishing the two subtypes. These findings highlight MD-pCASL as a valuable tool for PD subtyping and therapeutic monitoring.

## 1 Introduction

Parkinson’s disease (PD) is hypothesized to arise from a complex interplay between genetic and environmental factors; however, its precise etiology remains unclear. The most widely accepted pathological hallmark of PD is dopaminergic neurons degeneration in the substantia nigra pars compacta, which disrupts striatal dopamine regulation, thus ultimately leading to motor dysfunction (Kalia and Lang, 2015). These neurons are closely associated with the cerebral microvasculature; their degeneration reduces metabolic demand, thereby altering regional cerebral perfusion patterns (Kim et al., 2016). Recent neuroimaging studies have identified blood–brain barrier (BBB) disruption as a critical pathogenic feature in neurodegenerative mechanisms (Sweeney et al., 2018). In patients with PD, evidence of BBB dysfunction includes erythrocyte extravasation, protein accumulation within the striatum (Gray and Woulfe, 2015), thinning of the capillary endothelial layer, and widespread extraluminal immunoglobulin G (IgG) staining in the subthalamic nucleus (Pienaar et al., 2015). These findings suggest that PD might also represent a cerebrovascular disorder characterized by microvascular dysregulation (Nanhoe-Mahabier et al., 2009; Rane et al., 2020).

Arterial spin labeling (ASL), which is a noncontrast magnetic resonance imaging (MRI) modality for perfusion measurement, utilizes magnetically labeled blood water as an endogenous tracer. This technique quantifies cerebral blood flow (CBF) by applying radiofrequency pulses to magnetically invert the arterial blood proximal to the imaging region. However, conventional ASL methods have limitations to hemodynamic quantification owing to inter-individual variability and reliance on a single post-labeling delay (PLD), which may inaccurately represent heterogeneous blood flow velocities. To overcome these limitations, Cohen et al. (2020) pioneered an advanced multi-PLD ASL strategy termed multi-delay pseudo-continuous ASL (MD-pCASL), which enhances CBF accuracy by accounting for diverse arterial transit times (ATTs). This technique employs a short-burst radiofrequency pulse train to achieve high temporal resolution while maintaining an inversion efficiency comparable to that of continuous ASL. Additionally, MD-pCASL minimizes magnetization transfer effects and improves labeling efficiency, thereby yielding superior signal-to-noise ratios and broadening the clinical applicability of ASL. MD-pCASL quantifies two critical parameters: the ATT, which measures the duration of blood travel from the labeling plane to the imaging voxel, and ATT-corrected CBF, which provides a more precise estimate of capillary-level blood delivery rates by compensating for delayed arterial arrival.

Previous MRI studies have extensively documented neurovascular alterations in patients with PD. Wang et al. compared the diagnostic performance of conventional single-delay ASL and multi-delay ASL in PD cases and demonstrated that integrated models incorporating hemodynamic features from multi-delay ASL exhibited superior diagnostic utility. Compared with single-delay ASL, this approach offers detailed visualization of abnormal CBF and additional insights into ATT and cerebral blood volume (CBV), particularly in motor-associated regions (Wang et al., 2024). Similarly, Al-Bachari et al. (2017) found that patients with idiopathic PD exhibit diffuse ATT prolongation and focal CBF reduction. Notably, they were the first to report more widespread ATT prolongation in the tremor-dominant (TD) subtype than in the postural instability/gait difficulty subtype, despite observing no significant CBF differences between them.

Recent studies have validated MD-pCASL as a superior technique for assessing cerebrovascular disease, particularly for perfusion quantification (Yu et al., 2021; Luijten et al., 2023). However, the hemodynamic differences underlying the phenotypic divergence between the TD and akinetic–rigid-dominant (ARD) subtypes of PD remain poorly characterized. Elucidating these subtype-specific perfusion patterns might provide novel mechanistic insights into the clinical heterogeneity of PD, thereby bridging the gap between neurovascular pathophysiology and motor symptom manifestations. The current study aimed to investigate the differences in global cerebral hemodynamics between TD and ARD subtypes using MD-pCASL imaging and evaluate the diagnostic efficacy of multi-parameter MD-pCASL technique for motor subtypes.

## 2 Materials and methods

### 2.1 Study cohort and clinical data acquisition

We recruited patients with PD who were evaluated at the Department of Neurology at our hospital between 2018 and 2024. The inclusion criteria were: (1) diagnosis of PD according to the 2015 International Parkinson and Movement Disorder Society diagnostic criteria, (2) age range of 45–80 years, (3) Hoehn and Yahr (H-Y) stage ≤ 3, and (4) right-handedness. The exclusion criteria were: (1) comorbid psychiatric or neurological disorders other than PD, (2) intracranial organic lesions (e.g., tumors and traumatic injuries), (3) major systemic diseases, and (4) individuals with poor MRI image quality owing to noncompliance during scanning or contraindications for MRI.

Prior to MRI scanning, two senior neurologists specializing in movement disorders conducted comprehensive clinical assessments of the enrolled patients with PD. The evaluation protocol included (1) height and weight measurement to calculate body mass index, (2) documentation of disease duration, (3) disease severity quantification using the H-Y staging scale, (4) motor impairment severity assessment via the Unified Parkinson’s Disease Rating Scale Part III (UPDRS-III), (5) evaluation of cognitive deficits through the Mini-Mental State Examination (MMSE) and Montreal Cognitive Assessment (MoCA), (6) psychiatric comorbidity screening using the Self-Rating Anxiety Scale and Beck Depression Inventory, (7) characterization of sleep disorders with the REM Sleep Behavior Disorder Screening Questionnaire and Pittsburgh Sleep Quality Index, (8) constipation severity grading via the Constipation Severity Scale, and (9) detection of olfactory dysfunction using the PD-Specific Hyposmia Identification Card.

UPDRS-III and H-Y staging scores were assessed during the “ON” state, which is defined as evaluations conducted within 6 h after the last dose of levodopa or dopamine agonists. Following the methodology of Tang et al. (2021), phenotypic classification was determined by calculating the ratio between the mean tremor-related scores (items 20 and 21 of UPDRS-III) and mean rigidity-related scores (items 22, 27, and 31 of UPDRS-III). The patients were categorized as TD if the ratio exceeded 1.0, ARD if it was below 0.8, and mixed type if between 0.8 and 1.0. Mixed-type cases were excluded, thus resulting in a final cohort of 51 patients with PD (26 in the TD group and 25 in the ARD group). Additionally, 25 age-matched healthy controls (HC) were recruited. All participants provided written informed consent; the study protocol was approved by the hospital’s Ethics Committee.

### 2.2 Scanning devices and parameters

Imaging was performed using a 3.0 T Discovery™ MR750W MRI scanner (GE Healthcare, Chicago, IL, USA) equipped with a 19-channel head–neck combined coil. Foam padding was used to immobilize the head and minimize motion artifacts, whereas eye shields and earplugs were used to reduce sensory interference from ambient light and acoustic noise. All participants underwent two imaging sequences: 3D Fast Spoiled Gradient Echo (3D-FSPGR) and MD-pCASL. The parameters of 3D-FSPGR were: repetition time (TR) = 7.2 ms, echo time (TE) = 3.1 ms, matrix size = 256 × 256, field of view (FOV) = 256 mm × 256 mm, flip angle = 12°, number of excitations = 1, slice thickness = 1 mm with no interslice gap, and acquisition time = 4 min 55 s. The use of 3D-FSPGR yielded 128 contiguous axial slices covering the entire brain. The parameters of MD-pCASL were: TR = 6,046 ms; TE = 11.4 ms; FOV = 240 mm × 240 mm; spiral arms = 6; sampling points = 600; slice thickness = 4 mm (no gap, axial orientation, 36 slices); and PLDs = 1,000, 2,250, and 3,500 ms. The total scan time for MD-pCASL was 3 min 18 s.

### 2.3 ASL image preprocessing

Perfusion data were processed using MATLAB 2013b (MathWorks) with the SPM8 toolbox^1^ and the DPABI toolkit (Yan et al., 2016). The preprocessing pipeline included the following steps. First, motion correction was performed to eliminate translational and rotational artifacts, followed by quality control screening to exclude participants with excessive head motion (>2 mm translation or >2° rotation). Subsequently, a two-stage co-registration approach was applied: (1) linear registration of native-space perfusion images to their corresponding T1-weighted anatomical scans and (2) nonlinear normalization of T1 images to the Montreal Neurological Institute stereotactic space using diffeomorphic anatomical registration. Voxel-based resampling to a 2 mm × 2 mm × 2 mm isotropic resolution was implemented during spatial normalization. Data standardization was achieved by subtracting the global mean and dividing it by the standard deviation across all voxels. Spatial smoothing was then applied to CBF and ATT maps by using a 3-mm full-width-at-half-maximum Gaussian kernel. Finally, arterial CBV maps were computed as the product of the CBF and ATT, representing the volumetric delivery of labeled arterial blood from the tagging plane to the imaging voxel (Wang et al., 2013):


CBV=CBF×ATT

### 2.4 Statistical analysis

Statistical analyses were performed using SPSS (version 19.0; IBM Corp.). Normality and homogeneity of variance were assessed using the Shapiro–Wilk test and Levene’s test, respectively. Normally distributed continuous variables are expressed as mean ± standard deviation, with intergroup comparisons performed via independent samples *t*-test (two groups) or one-way analysis of variance (ANOVA; three groups). Nonnormally distributed continuous variables are reported as median (interquartile range) and analyzed using the Mann–Whitney U test (two groups) or Kruskal–Wallis H test (three groups). Categorical data are presented as counts (*n*), with between-group differences evaluated by Pearson’s chi-square (*χ*^2^) test. Statistical significance was set at *p* < 0.05 (two-tailed).

Statistical analyses of CBF and ATT maps were performed using MATLAB 2013b and the statistical module of the DPABI toolkit. One-way analysis of covariance (ANCOVA) was conducted to compare between the HC, ARD, and TD groups, with age, sex, height, weight, and years of education as covariates. Intergroup differences were evaluated using a two-sample *t*-test. Statistical significance was set at *p* < 0.005 at the voxel level and *p* < 0.05 at the cluster level, with Gaussian random field (GRF) correction applied to identify significant regional differences, which were then overlaid onto the standard Ch2 template. The anatomical localization of differential clusters was performed using the automated anatomical labeling 3 (AAL3) atlas with 170 parcellated regions (Rolls et al., 2020). Notably, the AAL3 atlas incorporates previously undefined subcortical nuclei involved in neuroimaging research, including PD-relevant deep gray matter structures such as the thalamus, nucleus accumbens, substantia nigra, ventral tegmental area, red nucleus, locus coeruleus, and raphe nuclei.

The CBF, CBV, and ATT in brain regions with differences in hemodynamic parameters between the ARD and TD groups were extracted, and *Z*-scores were converted. Thereafter, the stepwise regression method of binary logistic regression analysis was used to determine and distinguish the independent influencing factors of motor subtypes. Models were constructed; receiver operating characteristic (ROC) curves were drawn to evaluate the differential value of indicators with area under the curve (AUC).

### 2.5 Subtype-specific perfusion–clinical correlation profiling

Regionally distinct brain areas identified through group-level comparisons were converted into binary masks to define regions of interest (ROIs). The CBF, CBV, and ATT values corresponding to these ROIs were extracted for each participant cohort. Subsequently, Pearson’s and Spearman’s correlation analyses were conducted to assess the relationship between the hemodynamic parameters (CBF, CBV, and ATT) and clinical assessment scores across all subgroups. To correct for multiple comparisons, Bonferroni correction was applied, and *p* < 0.05 (after adjustment) was considered statistically significant.

## 3 Results

### 3.1 Socio-demographic and clinical characteristics

The HC, ARD, and TD groups comprised 25, 25, and 26 patients, respectively. No significant differences were found in the basic clinical data of general clinical features between groups (*p* > 0.05); Table 1 presents the detailed results.

**Table 1:** Basic characteristics of the participants.

Category	HC (*n* = 25)	ARD (*n* = 25)	TD (*n* = 26)	*P1*	*P2*
Age (y)	61.08 ± 6.37	64.60 ± 7.87	64.96 ± 7.64	0.123^a^	0.869^c^
Gender (M/F)	15/10	15/10	10/16	0.204^b^	0.124^b^
BMI	23.94 ± 3.27	25.10 ± 2.94	23.73 ± 3.04	0.248^a^	0.112^c^
Duration of illness (y)	—	4.48 ± 2.62	4.94 ± 3.44	—	0.593^c^
H-Y	—	2 (0.5)	2.08 ± 0.85	—	0.914^d^
UPDRS-III	—	27.12 ± 9.69	23.46 ± 14.75	—	0.302^c^
MoCA	—	22 (7.5)	19.81 ± 5.14	—	0.406^d^
SAS	—	48.04 ± 10.10	46.35 ± 8.85	—	0.527^c^
BDI	—	9 (16)	8.5 (15.75)	—	0.955^d^
RBDSQ	—	9 (18)	8.5 (23.25)	—	0.624^d^
PSQI	—	5 (5)	7.5(7.5)	—	0.072^d^
CSS	—	8.44 ± 5.48	6.31 ± 6.18	—	0.199^c^
Olfactory	—	5 (3)	5.35 ± 2.37	—	0.505^d^

P1: comparisons among the HC, ARD, and TD groups; P2: comparisons between the ARD and TD groups. HC, healthy control; ARD, akinetic-rigid dominant; TD, tremor-dominant; BMI, body mass index; H-Y, Hoehn and Yahr; UPDRS-III, Unified Parkinson’s Disease Rating Scale Part III; MoCA, Montreal Cognitive Assessment; SAS, Self-Rating Anxiety Scale; BDI, Beck Depression Inventory; RBDSQ, REM Sleep Behavior Disorder Screening Questionnaire; PSQI, Pittsburgh Sleep Quality Index; CSS, Constipation Severity Scale.

a ANOVA.

b Chi-square test.

c Two-sample *t*-test.

d Mann–Whitney U test.

### 3.2 Voxel-based analysis of CBF, CBV, and ATT differences

**Figure 1:**
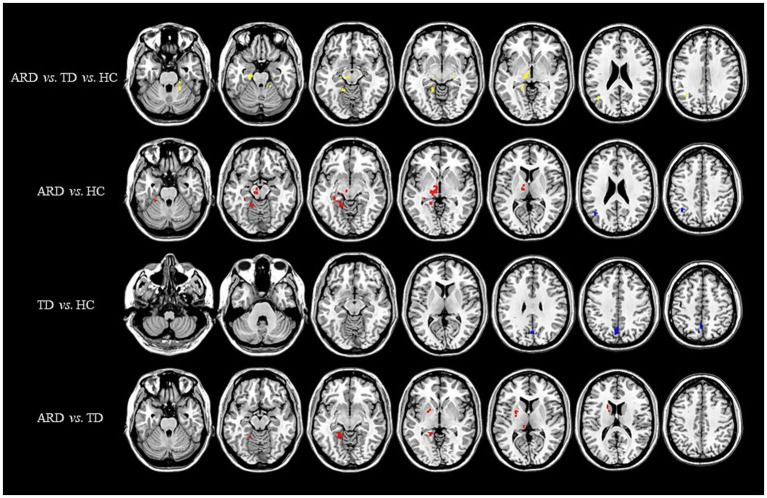
Distribution of CBF with significant intergroup differences. Regions with increased CBF are indicated in red, whereas regions with decreased CBF are shown in blue. CBF, cerebral blood flow; ARD, akinetic-rigid dominant; TD, tremor-dominant; HC, healthy control.

**Figure 2:**
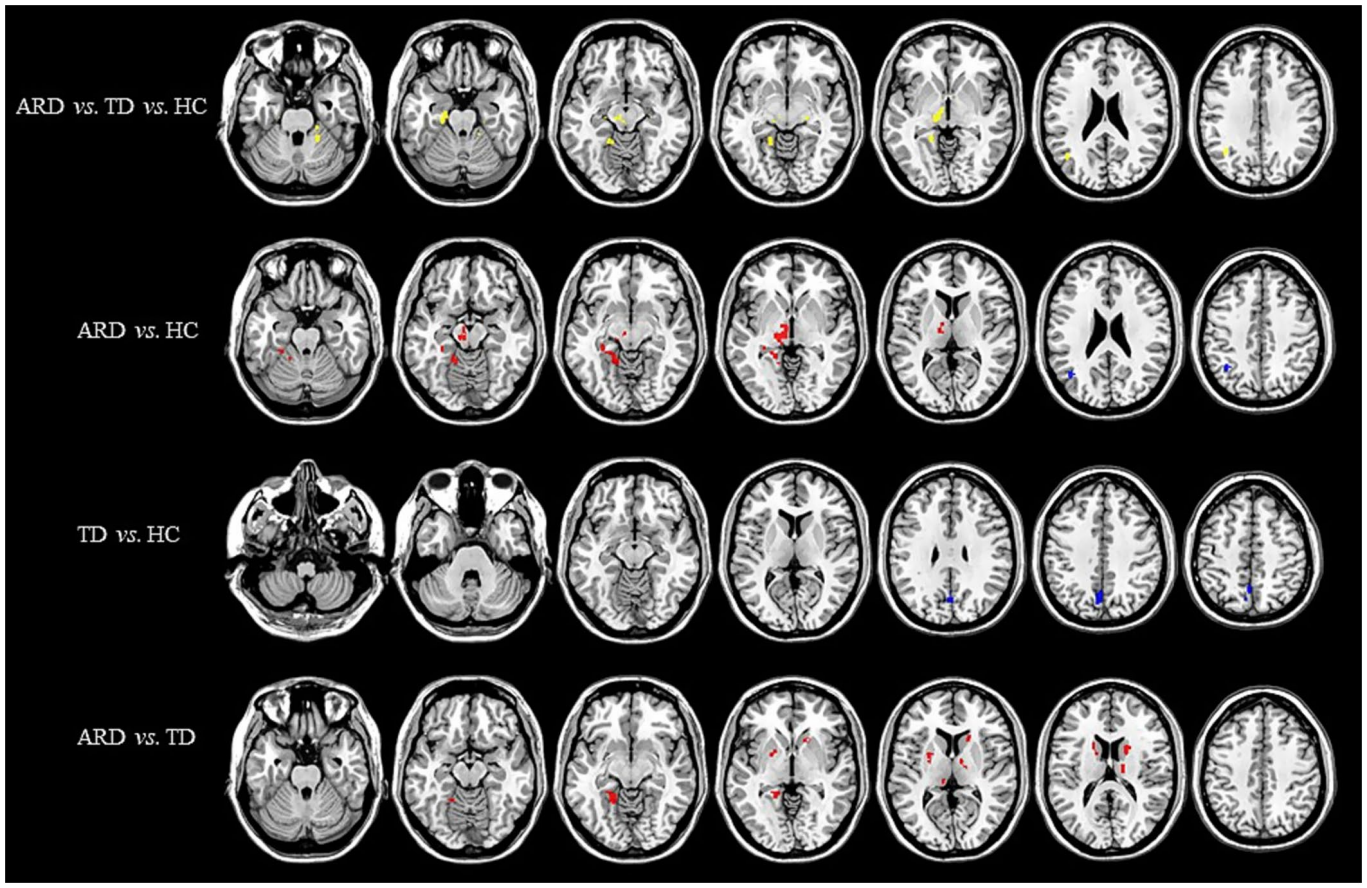
Distribution of CBV with significant intergroup differences. Regions with increased CBV are indicated in red, whereas regions with decreased CBV are indicated in blue. CBV, cerebral blood volume; ARD, akinetic-rigid dominant; TD, tremor-dominant; HC, healthy control.

**Figure 3:**
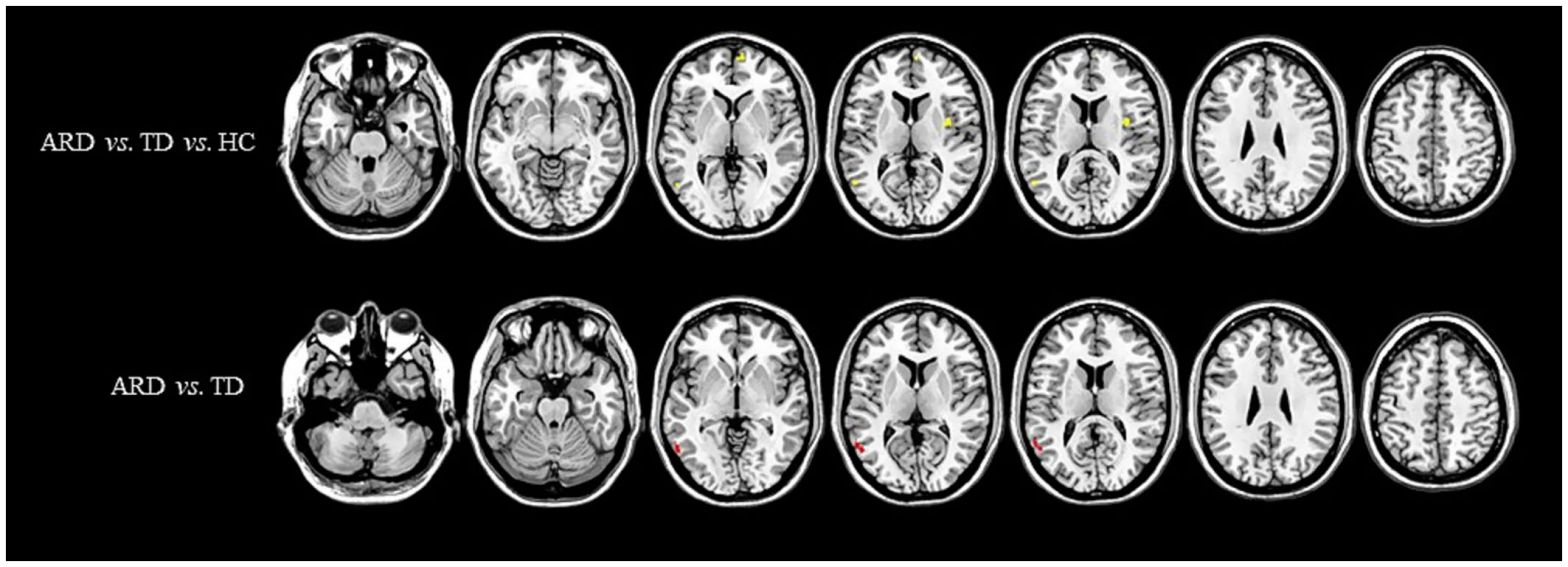
Distribution of ATT with significant intergroup differences. Regions with increased ATT are indicated in red, whereas regions with decreased ATT are indicated in blue. ATT, arterial transit time; ARD, akinetic-rigid dominant; TD, tremor-dominant; HC, healthy control.

Voxel-based whole-brain analysis of CBF and CBV revealed significant intergroup differences (GRF correction: *p* < 0.005 at the voxel level and *p* < 0.05 at the cluster level), as illustrated in Figures 1–3 and Tables 2–4.

**Table 2:** Details of CBF differences between groups based on AAL3 brain atlas.

Group	AAL3	Peak point MNI coordinates			Peak intensity	Cluster size
		X	Y	Z		
ARD/TD/HC	Left thalamus, left substantia nigra and red nucleus	−14	−20	−4	5.44751	353
	Left angular gyrus, left inferior parietal lobule	−38	−56	32	10.9401	103
	Left hippocampus, left parahippocampal gyrus	−22	−18	−24	14.4006	101
	Left lingual gyrus, left cerebellar lobules IV-V, left fusiform gyrus	−16	−46	−12	12.3035	85
	Right cerebellar lobules IV-V, right lateral geniculate nucleus	22	−44	−28	11.6105	67
ARD > HC	Left thalamus, left substantia nigra and red nucleus	−14	−20	−4	5.42737	351
	Left lingual gyrus, left fusiform gyrus, left hippocampus, left parahippocampal gyrus, left cerebellar lobules IV-V	−16	−46	−12	5.08916	258
ARD < HC	Left angular gyrus, left inferior parietal lobule	−42	−62	26	−4.51184	244
TD < HC	Left precuneus, left cuneus	−4	−66	42	−4.70732	205
ARD > TD	Left putamen, left caudate nucleus, left globus pallidus	−24	0	8	3.96883	198
	Left lingual gyrus, left hippocampus, left medial ventral posterior thalamic nucleus, left parahippocampal gyrus, left fusiform gyrus	−18	−44	−6	5.26374	193

CBF, cerebral blood flow; AAL3, automated anatomical labeling 3; MNI, Montreal Neurological Institute; ARD, akinetic-rigid dominant; TD, tremor-dominant; HC, healthy control.

**Table 3:** Details of CBV differences between groups based on AAL3 brain atlas.

Group	AAL3	Peak point MNI coordinates			Peak intensity	Cluster size
		X	Y	Z		
ARD/TD/HC	Left thalamus, left substantia nigra and red nucleus	−12	−20	−4	10.4277	132
	Left angular gyrus, left inferior parietal lobule	−38	−56	32	11.0462	106
	Left hippocampus, left parahippocampal gyrus	−22	−18	−24	14.316	101
	Left lingual gyrus, left cerebellar lobules IV-V, left fusiform gyrus	−16	−46	−12	12.3563	87
	Right cerebellar lobules IV-V, right lateral geniculate nucleus	22	−44	−28	11.4537	60
ARD > HC	Left thalamus, left substantia nigra and red nucleus	−14	−20	−4	5.44751	353
	left lingual gyrus, left fusiform gyrus, left hippocampus, left parahippocampal gyrus, and left cerebellar lobules IV-V	−16	−46	−12	5.13888	280
ARD < HC	Left angular gyrus, left inferior parietal lobule	−42	−62	26	−4.56736	240
TD < HC	Left precuneus, left cuneus	−4	−66	42	−4.73119	198
ARD > TD	Right caudate nucleus, right ventral thalamic nucleus	16	18	2	5.00036	213
	Left putamen, left caudate nucleus, left globus pallidus	−24	0	8	3.96401	187
	Left lingual gyrus, left hippocampus, left medial ventral posterior thalamic nucleus, left parahippocampal gyrus, left fusiform gyrus	−18	−44	−6	5.23025	192

CBV, cerebral blood volume; AAL3, automated anatomical labeling 3; MNI, Montreal Neurological Institute; ARD, akinetic-rigid dominant; TD, tremor-dominant; HC, healthy control.

**Table 4:** Details of ATT differences between groups based on AAL3 brain atlas.

Group	AAL3	Peak point MNI coordinates			Peak intensity	Cluster size
		X	Y	Z		
ARD/TD/HC	Right insula	38	0	12	17.301	46
	Left middle temporal gyrus	−54	−62	10	9.377	45
	Left medial superior frontal gyrus	10	64	0	11.0167	43
ARD >TD	Left middle temporal gyrus	−58	−60	−4	4.40829	139

ATT, arterial transit time; AAL3, automated anatomical labeling 3; MNI, Montreal Neurological Institute; ARD, akinetic-rigid dominant; TD, tremor-dominant; HC, healthy control.

One-way ANOVA identified statistically distinct CBF and CBV patterns among the HC, ARD, and TD groups in the following regions: left thalamus, substantia nigra–red nucleus complex, angular gyrus, inferior parietal lobule, hippocampus, parahippocampal gyrus, lingual gyrus, cerebellar lobules IV/V, fusiform gyrus, right cerebellar lobules IV–V, and lateral geniculate nucleus. There were statistically significant differences in the ATT patterns of the right insula, left middle temporal gyrus and left medial superior frontal gyrus among the three groups.

Two-sample *t*-test comparisons demonstrated that, relative to the HC group, the ARD group exhibited elevated CBF and CBV in the left thalamus, substantia nigra–red nucleus complex, lingual gyrus, fusiform gyrus, hippocampus, parahippocampal gyrus, and cerebellar lobules IV/V, whereas reduced CBF and CBV were observed in the left angular gyrus and inferior parietal lobule. The TD group showed diminished CBF and CBV in the left precuneus and cuneus compared with the HC group.

The ARD group displayed significantly higher CBF and CBV than did the TD group in the left putamen, caudate nucleus, globus pallidus, lingual gyrus, medial ventral posterior thalamic nucleus, hippocampus, parahippocampal gyrus, and fusiform gyrus. Notably, the ARD group displayed significantly higher CBV than did the TD group in the right caudate nucleus, ventral thalamic nucleus. In addition, the ATT in the left middle temporal gyrus of the ARD group was prolonged compared with that of the TD group.

### 3.3 Binary logistic regression analysis and ROC curve

**Figure 4:**
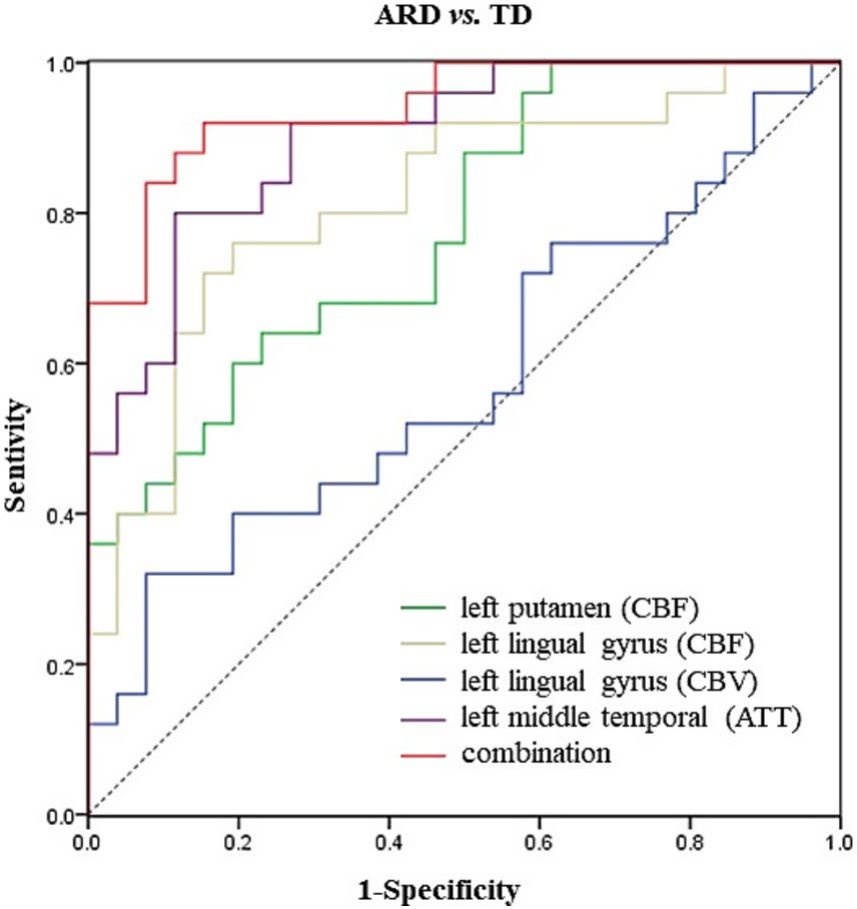
ROC curve of differential CBF, CBV and ATT values for ARD and TD Parkinson’s disease.

The analysis results of the ARD group and the TD group showed that when ARD was diagnosed as positive, changes in CBF, CBV and ATT in some brain regions were considered risk factors. The specific results are listed in Tables 2–5. The ROC curve showed that the AUC of the left putamen CBF was 0.780, optimal critical value was 0.409, sensitivity was 64.0%, and specificity was 76.9%. The AUC of the CBF of the left lingual gyrus was 0.817, optimal critical value was 0.568, sensitivity was 76.0%, and specificity was 80.8%. The AUC of CBV of the left lingual gyrus was 0.582, optimal critical value was 0.243, sensitivity was 32.0%, and specificity was 92.3%. The AUC of ATT in the left medial temporal gyrus was 0.900, optimal critical value was 0.685, sensitivity was 80.0%, and specificity was 88.5%. It is worth noting that the comprehensive AUC of each index was 0.942, optimal critical value was 0.766, sensitivity was 92%, and specificity was 84.6%. Figure 4 shows the specific results.

**Table 5:** Binary Logistic regression analysis of ARD group and TD group.

Parameter	Factor	B	S. E	*P*	OR	95% CI of OR
CBF	Left putamen	4.245	1.791	0.018	69.757	2.084 ~ 2335.352
	Left lingual gyrus	2.968	1.108	0.008	19.459	2.218 ~ 170.707
	constant	0.615	0.582	0.291	1.849	–
CBV	Left lingual gyrus	4.781	2.405	0.047	119.230	1.070 ~ 13290.973
	constant	0.942	0.467	0.044	2.566	–
ATT	Left middle temporal gyrus	6.791	1.835	0.0002	889.454	24.405 ~ 32416.947
	constant	−2.378	0.738	0.001	0.093	–

CBF, cerebral blood flow; CBV, cerebral blood volume; ATT, arterial transit time; ARD, akinetic-rigid dominant; TD, tremor-dominant.

### 3.4 Correlation analysis

ROI analyses were performed on different brain regions to evaluate the Pearson’s correlations between cerebral hemodynamic parameters (CBF/CBV) and clinical assessments, with family wise error rates adjusted for using the Bonferroni correction (*p* < 0.05).

**Figure 5:**
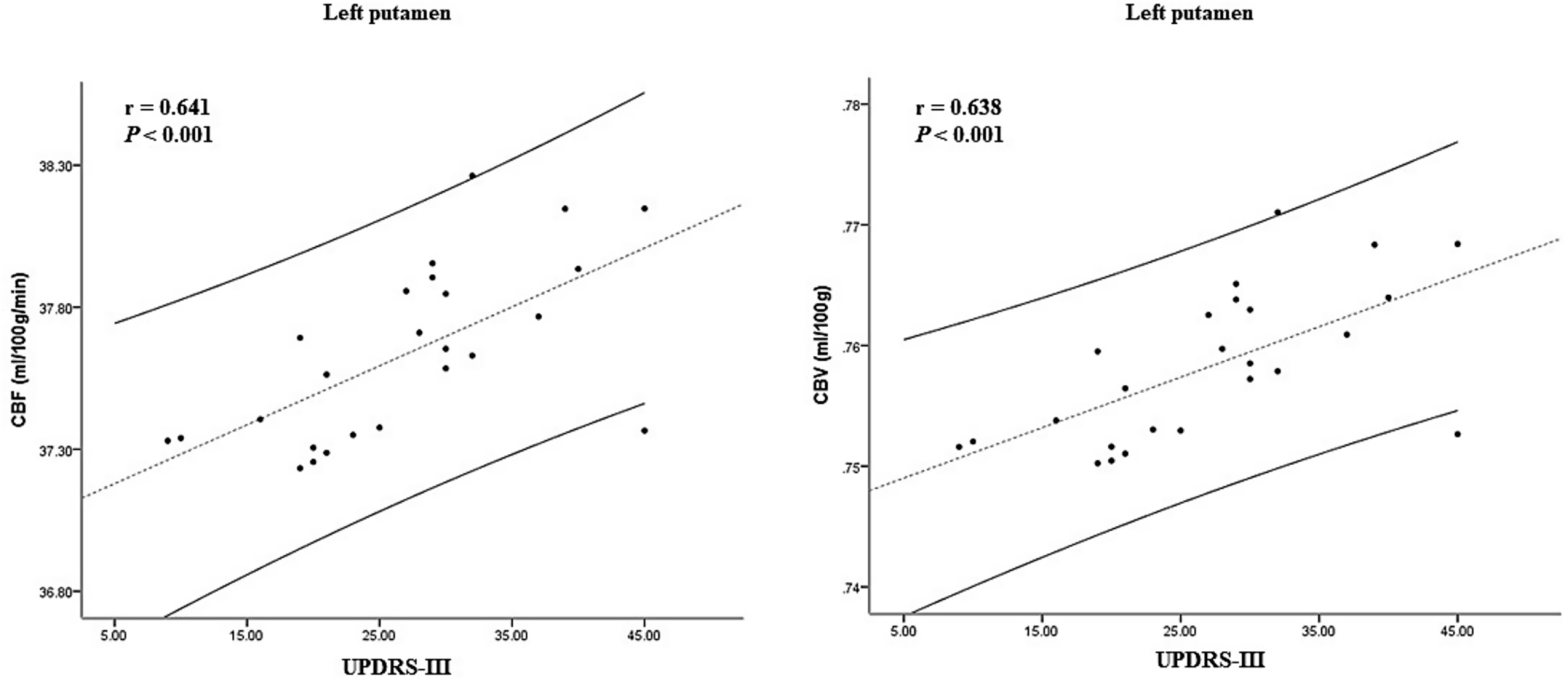
Correlation between CBF, CBV, and clinical scale score in the ARD group. CBF, cerebral blood flow; CBV, cerebral blood volume; ARD, akinetic-rigid dominant.

**Figure 6:**
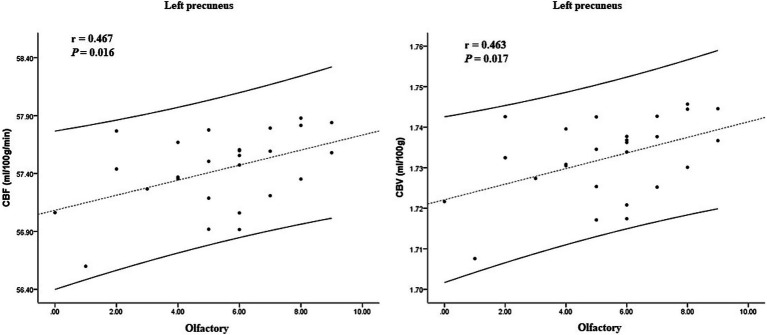
Correlation between CBF, CBV, and clinical scale score in the TD group. CBF, cerebral blood flow; CBV, cerebral blood volume; TD, tremor-dominant.

In the ARD group, the CBF and CBV values of the left putamen and its surrounding nuclei positively correlated with the UPDRS-III score (*r* = 0.641, *p* < 0.001; *r* = 0.638, *p* < 0.001), the specific results are shown in Figure 5. In the TD group, the CBF and CBV values of the left precuneus and cuneus positively correlated with the olfactory score (*r* = 0.467, *p* = 0.016, *r* = 0.463, *p* = 0.017), as shown in Figure 6.

## 4 Discussion

This study employed the novel MD-pCASL technique to comprehensively assess CBF, ATT, and CBV values in the TD, ARD, and HC groups without contrast administration. Our findings revealed that the ARD group exhibited elevated CBF and CBV in multiple left-hemispheric cortical regions compared with the HC group, whereas no hyperperfused areas were identified in the TD group. Furthermore, direct comparisons between the ARD and TD subgroups showed significantly higher perfusion levels in the ARD group, particularly in the left hippocampal formation and basal ganglia nuclei. Notably, our results showed marked hemispheric lateralization, with the most significant differences localized in the left cerebral hemisphere, a phenomenon potentially linked to predominantly right-sided motor symptom onset. This observation aligns with the seminal work of Shang et al. (2021), which posited that lateralized motor manifestations in patients with PD might reflect asymmetrical regional and interregional CBF abnormalities, thereby underscoring the necessity for stratifying PD subtypes based on motor laterality patterns.

Wang et al. compared the hemodynamic abnormalities in the brain of patients with PD on multiple delayed arterial spin labeling and the traditional single delayed arterial spin labeling technique. They found that the corrected CBF demonstrated better classification performance than did the uncorrected CBF. Moreover, the AUC of both CBV and ATT was relatively high. The comprehensive model emphasizing the hemodynamic characteristics of MDASL provides better performance for PD diagnosis (Wang et al., 2024). However, this study did not conduct a classification discussion about PD and ignored the influence of subtype heterogeneity. Based on this, our study demonstrated the different hemodynamic characteristics among the motor subtypes. The elevated CBF observed in the ARD subgroup suggests localized BBB hyperpermeability and greater neurovascular unit disruption (Li et al., 2020). Hippocampal formation—a hub for memory consolidation, spatial navigation, and emotional modulation—exhibited hyperperfusion in ARD cases, thus potentially contributing to its accelerated cognitive decline compared with the TD subtype (Wickremaratchi et al., 2011). Postmortem analyses of brains from patients with PD have revealed perivascular fibrinogen/fibrin (Gray and Woulfe, 2015), IgG (Pienaar et al., 2015), and hemosiderin (Loeffler et al., 1995) deposits within the striatal vasculature, which are indicative of chronic BBB breakdown. Al-Bachari et al. (2017) reported that the prolongation of ATT in the TD group was more diffuse. However, no differences in CBF among subtypes were found. Our research results have been supplemented in terms of CBF/CBV. Al-Bachari et al. (2017) also reported basal ganglia hyperperfusion and hypothesized that the exacerbated nigrostriatal pathway dysfunction in patients with ARD may drive this phenomenon, in contrast to the compensatory downstream neuromodulatory adaptations in those with TD (Zaidel et al., 2009). Notably, subthalamic nucleus deep brain stimulation–mediated tremor amelioration may involve microvascular remodeling (Sweeney et al., 2018), further implicating neurovascular crosstalk in PD symptomatology.

Pharmacologically, levodopa, which is the first-line therapy for PD, may influence basal ganglia perfusion. At therapeutic doses, levodopa is converted to dopamine by preserved dopaminergic neurons in the putamen, followed by a dysregulated release due to deficient autoreceptor feedback (Politis et al., 2014). The vasodilatory properties of dopamine enhance the regional CBF (Iadecola, 1998), potentially facilitating BBB-penetrant drug delivery. Pelizzari et al. (2019) identified positive correlations between UPDRS-III scores and CBF in the sensorimotor basal ganglia networks, a finding corroborated and extended by our study. Specifically, ARD exhibited stronger correlations with deep basal ganglia nuclear perfusion and motor severity, thus confirming the pivotal role of striatopallidal degeneration in ARD pathogenesis and progression.

This study did not identify significant differences in ATT between PD subgroups (ARD/TD) and HC. However, the ARD subgroup exhibited significantly prolonged ATT in the left middle temporal gyrus compared with the TD subgroup. Notably, studies on acute stroke have proposed that ATT prolongation may reflect preserved perfusion (Wolf et al., 2014) or collateral compensation (Zaharchuk, 2011), both mechanisms that warrant further investigation in neurodegenerative contexts. In addition, regression analysis and ROC curve analysis in this study showed that the combined indicators of CBF, CBV, and ATT had the best diagnostic efficacy in identifying PD subtypes. This preliminarily indicates that multi-parameter blood flow indicators can be used as effective imaging biological markers for distinguishing between motor subtypes.

P-glycoprotein, which is a critical cellular defense mechanism against neurotoxicant accumulation in the brain (Zhao et al., 2015), has been shown via positron emission tomography (PET) imaging to exhibit reduced activity in the midbrain of patients with PD, which is indicative of BBB dysfunction (Kortekaas et al., 2005). Previous quantitative PET studies have documented widespread cortical hypoperfusion and hypometabolism in the frontal–parietal–occipital regions of PD cohorts (Borghammer, 2012). Our findings further demonstrated that the ARD subgroup exhibited decreased CBF and CBV in the left angular gyrus and inferior parietal lobule compared with the HC group, with these hemodynamic reductions negatively correlating with MoCA and MMSE scores; this pattern is exacerbated by comorbid vascular pathologies and risk factors that are known to accelerate motor and cognitive decline in PD (Malek et al., 2016). Notably, diminished cerebral perfusion has also been reported in patients with mild cognitive impairment and often precedes structural atrophy (Kisler et al., 2017).

By contrast, the TD group showed reduced CBF and CBV in the left precuneus and cuneus, which are regions that are positively associated with olfactory function scores. Olfactory dysfunction, a hallmark prodromal feature of neurodegenerative diseases that precedes cognitive deficits (Murphy, 2019; Slabik and Garaschuk, 2023), persists throughout the disease course of TD-PD and may even antedate motor symptom onset (Wang et al., 2022). Although the cuneus and precuneus primarily contribute to visuospatial processing, their involvement in olfactory function may arise from visuo-olfactory integration or indirect modulation via default mode network pathways. Huang et al. (2024) investigated the neuroimaging mechanisms underlying olfactory regulation in patients with cerebral small vessel disease and similarly identified reduced regional homogeneity and functional connectivity in the left cuneus in those with PD, which correlated positively with olfactory performance.

This study has a few limitations that warrant consideration. First, the modest sample size necessitates the validation of statistical effects through larger multicenter cohorts in future investigations. Second, although MD-pCASL effectively mapped perfusion heterogeneity between TD and ARD groups, its mechanistic links to underlying neuropathological substrates—such as *α*-synuclein propagation or neurovascular unit degeneration—require further exploration in animal models or hemodynamic phantom studies. Third, our classification based on motor dysfunction phenotypes might have limited detection of nonmotor symptoms associated with cerebral perfusion, thus underscoring the need for transdiagnostic biomarkers in future PD subtyping frameworks. Fourth, the lack of standardized medication washout protocols (e.g., levodopa or dopamine agonists) might have obscured symptom severity or perfusion responses, a confounder that should be adjusted for in future longitudinal designs.

In conclusion, the MD-pCASL technology accurately identifies the specific change patterns of cerebral blood perfusion of TD and ARD types in patients with PD by optimizing multi-parameter hemodynamic detection. At the same time, it also confirms the clinical application potential of the MD-pCASL technology in the early differential diagnosis, subtype classification and disease course monitoring for PD. It is expected to promote the practical application of precision medicine in the field of neurodegenerative diseases.

## Data Availability

The original contributions presented in the study are included in the article/supplementary material, further inquiries can be directed to the corresponding authors.

## Footnotes

^1^ http://www.fil.ion.ucl.ac.uk/spm/
